# Student background, admission routes, and academic success: a structural mediation analysis

**DOI:** 10.1186/s12909-026-09068-z

**Published:** 2026-03-24

**Authors:** Stefanos A. Tsikas, Martin Kieca

**Affiliations:** Hannover Medical School (MHH), Dean of Studies Office – Academic Controlling, Carl-Neuberg-Straße 1, Hannover, D-30625 Germany

**Keywords:** Academic success, Student selection, Medical education, Medical students, Socioeconomic status, Structural equation modelling, Mediation analysis

## Abstract

**Background:**

Medical school admissions increasingly seek to balance cognitive merit with broader notions of potential and diversity. Yet, the extent to which different selection routes predict academic success, and how social background shapes these pathways, remains debated. Using linked administrative and survey data from 644 students at a large German medical school, we examined how sociodemographic background, prior educational trajectories, and parental characteristics influence both admission routes and early academic performance.

**Methods:**

We estimated a structural equation model (SEM) with observed variables to represent temporally ordered educational pathways linking background characteristics, school performance, admission routes, and academic success. Direct and indirect effects were quantified using mediation analysis, including the proportion mediated to assess the relative importance of indirect pathways.

**Results:**

Indicators of cognitive achievement in secondary education emerged as the strongest and most consistent predictors of medical school performance. Parental education and school achievement facilitated direct access to medical school via more favourable educational trajectories. Gender had no meaningful indirect or direct influence on admission routes or performance. Admission routes that placed greater weight on non-cognitive criteria were independently associated with lower exam performance, even after accounting for prior achievement.

**Discussion:**

Our findings indicate that within the highly selected and academically rather homogeneous population of medical students, effects of demographic and socioeconomic background on study success and student selection operate predominantly through earlier educational pathways. Inequalities in opportunity arise primarily before university admission, not within it. For medical schools, this implies that fairness is best promoted not by compensating for background through increasingly complex non-cognitive selection tools, but by maintaining transparent, evidence-based admission systems anchored in criteria with demonstrated predictive validity for study success.

**Supplementary Information:**

The online version contains supplementary material available at 10.1186/s12909-026-09068-z.

## Introduction

### Background

University admission procedures aim to identify applicants most likely to succeed academically, typically defined as completing their studies with strong grades and within a reasonable timeframe. In medicine, where education is inherently vocational and graduates assume responsibility for patient care, rigorous selection is especially crucial. Yet while admissions should ideally foster not only cognitive competence but also professional attributes, defining, and even more so measuring, what makes a “good” doctor remains challenging [1, 2]. 

In most countries, secondary school attainment forms the foundation of university entry. Measures of prior academic performance, such as Abitur grades, GPA, or A-levels, remain among the strongest predictors of academic and occupational outcomes [3–8]. 

In medicine, these indicators are often supplemented (or partially replaced) by aptitude tests [9–12], interviews [13–17], personality assessments [18–20], or evidence of prior medical experience [21–24]. 

Beyond individual aptitude, academic performance is shaped by social context. Socioeconomic status (SES) and parental education exert consistent and often intergenerational effects on educational outcomes [25–31]. In Germany, for example, school grades determine whether students enter medical school directly or only after a waiting period, often bridged by vocational training. These pathways influence not only age at enrolment but also how studies are financed, e.g. whether through family support or employment, both of which have implications for focus and time to completion.

An effective and unbiased selection system should prioritize criteria with proven predictive validity for study success. Academic performance, aptitude, and relevant prior experience should facilitate access and achievement, while demographic characteristics such as gender, ethnicity, or migration background should not directly affect outcomes. Still, indirect influences are well documented: girls often outperform boys at school [32–37], and students from educated or native-born families typically benefit from greater academic support and resources [26, 38–43]. 

What remains less understood, particularly in medicine, is how these individual and contextual factors interact with each other and with complex admission regimes to shape academic success. Selection systems themselves may have incremental effects if they place disproportionate weight on criteria with limited predictive validity or emphasize intangible attributes of uncertain relevance to future medical competence [44–46]. 

### Study objectives

This study brings these strands together by modelling both direct and indirect pathways linking sociodemographic background, cognitive performance, and study success. Rather than treating individual characteristics as independent predictors, our analytical framework explicitly conceptualizes them as temporally ordered and causally connected components of educational trajectories, allowing for multiple sequential mediation processes. Using structural equation modelling and mediation analysis, we test whether (1) predictors of academic success are reflected in different admission routes, (2) whether social background and status affect outcomes directly or through intermediate educational and life-course related characteristics, and (3) whether selection systems exert independent effects on performance by emphasizing non-cognitive criteria. This approach allows total associations to be decomposed into direct and indirect effects and enables us to assess the extent to which admission routes function as mediators within a broader system of linked individual characteristics.

### Expected results

Based on prior research on predictors of academic performance in medical education and on the structure of our analytical model, we formulate the following expectations:

First, we expect cognitive criteria assessed prior to admission, most notably secondary school achievement, to show a strong, direct, and positive association with academic success during medical training. These variables have repeatedly demonstrated predictive validity for study performance and progression. Consequently, in admission routes that rely heavily on such cognitive indicators, we also expect these characteristics to exert a substantial influence on admission probabilities. In this sense, admission routes can be understood as mechanisms that translate academically relevant individual characteristics into selection outcomes.

Second, we expect that socio-demographic characteristics such as gender, parental education, or migration background do not exert a direct effect on academic success once prior academic achievement and educational pathways are taken into account. From a substantive perspective, these characteristics lack intrinsic academic relevance. Accordingly, under an admission regime that is closely aligned with cognitively predictive criteria, these background characteristics should not independently affect either study success or admission outcomes.

However, we further expect that this pattern may change in admission routes that place less emphasis on cognitively relevant criteria and instead prioritize alternative indicators such as prior vocational training, motivational statements, or signals of social or personal competencies. To the extent that such criteria are weakly related (or unrelated) to subsequent academic performance, admission procedures may acquire an independent effect on study success. In such cases, admission routes may not merely mediate academically relevant characteristics but may introduce adverse selection effects, potentially disadvantaging academic outcomes.

A key feature of our analytical approach is the explicit modelling of individual characteristics as part of interconnected causal chains rather than as isolated, independent predictors of admission and academic success. We assume that parental background characteristics shape earlier educational trajectories, including school type attended and school performance. Strong school achievement facilitates direct access to medical school, whereas weaker performance may lead individuals to alternative educational pathways, often involving vocational training in the health sector, before entering medical education at a later stage. As a result, students admitted through different routes may systematically differ in age, life experience, family responsibilities, and economic constraints.

In line with this perspective, we expect that the influence of background characteristics on academic success is predominantly indirect and operates through sequential mediation pathways involving educational achievement, admission routes, and life-course-related characteristics. We further expect these indirect effects to be more pronounced in admission systems that strongly emphasize cognitive selection criteria, as academically relevant characteristics are more consistently transmitted through both admission and subsequent academic performance. These expectations motivate our focus on decomposing total effects into direct and indirect components and on interpreting admission routes as embedded within a broader system of mediated relationships rather than as a single, isolated mechanism.

## Methods

### Data and setting

Data were drawn from 644 medical students at Hannover Medical School (MHH) who participated in the baseline survey of the *Hannover Screening of Study Motivation* (HSM) between 2021 and 2024, had valid M1 final grades (ranging from A to C), and were admitted through one of the three main admission quotas considered in this study (see the “Study variables” section below). Students admitted via small “pre-quota” categories (e.g., international applicants, military physicians, or hardship cases) were excluded due to the heterogeneity of these groups and the distinct selection criteria applied.

The HSM has been conducted annually as a panel survey since 2021 and collects information on study motivation, sociodemographic background, personality traits, study progress, and career aspirations (Tsikas and Fischer: Dimensions of medical students’ motivation and their stability over time: longitudinal evidence from a German panel data survey, submitted). Most sociodemographic variables are collected at baseline only, as they refer to fixed characteristics or past events. The average response rate of the HSM baseline surveys was approximately 19%. Comparisons with administrative student statistics indicate that the HSM sample is broadly representative of the overall student population at MHH with respect to key characteristics such as age distribution, school background, and academic performance.

Within the HSM, an informed consent clause was incorporated, requiring participants to agree to the use of their responses and to the merger with student statistics and exam results. The informed consent and study regulations governing the use of personal data for evaluation/research and quality assurance purposes (in particular Sect.  14, para. 1–5 ‘*MHH Immatrikulationsordnung*’ and Sect.  17, para. 3 NHG (Higher Education Act in Lower Saxony, Germany)) rendered a separate approval by an ethics committee unnecessary. Participation and withdrawal in the questionnaire were voluntary at any given point. The research presented here is in accordance with the Helsinki Declaration.

### Study variables

Our primary outcome measure for academic performance is the overall score in the first section of the German medical licensing examination (M1). In the German system, M1 grades range from 1 (“A”) to 4 (“D”). At Hannover Medical School (MHH), unlike in the national standard curriculum, M1 is not administered as a single high-stakes examination at the end of the preclinical phase but is derived from graded module examinations across the first two years of study [47]. These assessments consist predominantly of multiple-choice tests (MC), complemented by several oral examinations and an Objective Structured Clinical Examination (OSCE) in diagnostic methods. Most students pass M1 with a grade of 3 (“C”) or better; the few observations with grade D or failure were considered outliers and excluded from the analysis.

We analyse students admitted via the three principal admission routes used in Germany: 30% of places are centrally allocated through the *Abiturbestenquote* (“top school leavers”, AQ), awarded to applicants with the best secondary school grades. Until 2020, a further 20% were allocated via the waiting-time quota (WQ), in which applicants were ranked by Abitur grade and admitted after extended waiting periods. During this time, most applicants completed vocational training in medical fields such as nursing or emergency medical services. This route has since been gradually replaced by the “special aptitude quota” (10% of places), which at MHH is reserved for applicants with professional medical experience and ranked according to performance on a medical school aptitude test. Because core characteristics such as school grades, vocational training, and age at enrolment are highly similar under both schemes [48], we pool these groups under the term WQ. The remaining 60% of places are allocated through university-specific procedures. At MHH, these currently combine Abitur grades, aptitude test results, and non-cognitive criteria such as prior medical or voluntary service experience. Until 2019, structured interviews were also used [49, 50]. Other German medical schools employ multiple mini-interviews [51–53] or personality-based assessments [7, 20, 54–56]. Overviews of German medical school admissions and recent reforms are provided by Schwibbe et al. [54] and Tsikas & Fischer [48]. 

Further, we include several individual characteristics that predate admission and study outcomes, but influence both directly and indirectly (see the study design below): the type of secondary school attended (Gymnasium versus other school types), the Abitur grade (ranging from 1.0 = highest to 4.0 = lowest passing grade), and whether students completed vocational training in a medical field prior to enrolment. In addition to age at enrolment and gender, we observe parental background characteristics, including parental nativity (mother and father born in Germany or abroad) and parental education (mother and father holding a university degree or not). Finally, we include information on how students primarily finance their studies. We use a dichotomous indicator distinguishing financial support by the family from other sources such as paid employment or student loans. This variable serves as a proxy for the socioeconomic resources available to the student and their family but is measured only after enrolment and therefore does not enter the admission stage of the model.

Information on parental background, prior vocational training, and study financing was obtained from the HSM. Administrative sources supplied information on academic outcomes, admission quotas, Abitur grade and school type, as well as age and gender.

### Study design and conceptual model

**Fig. 1:**
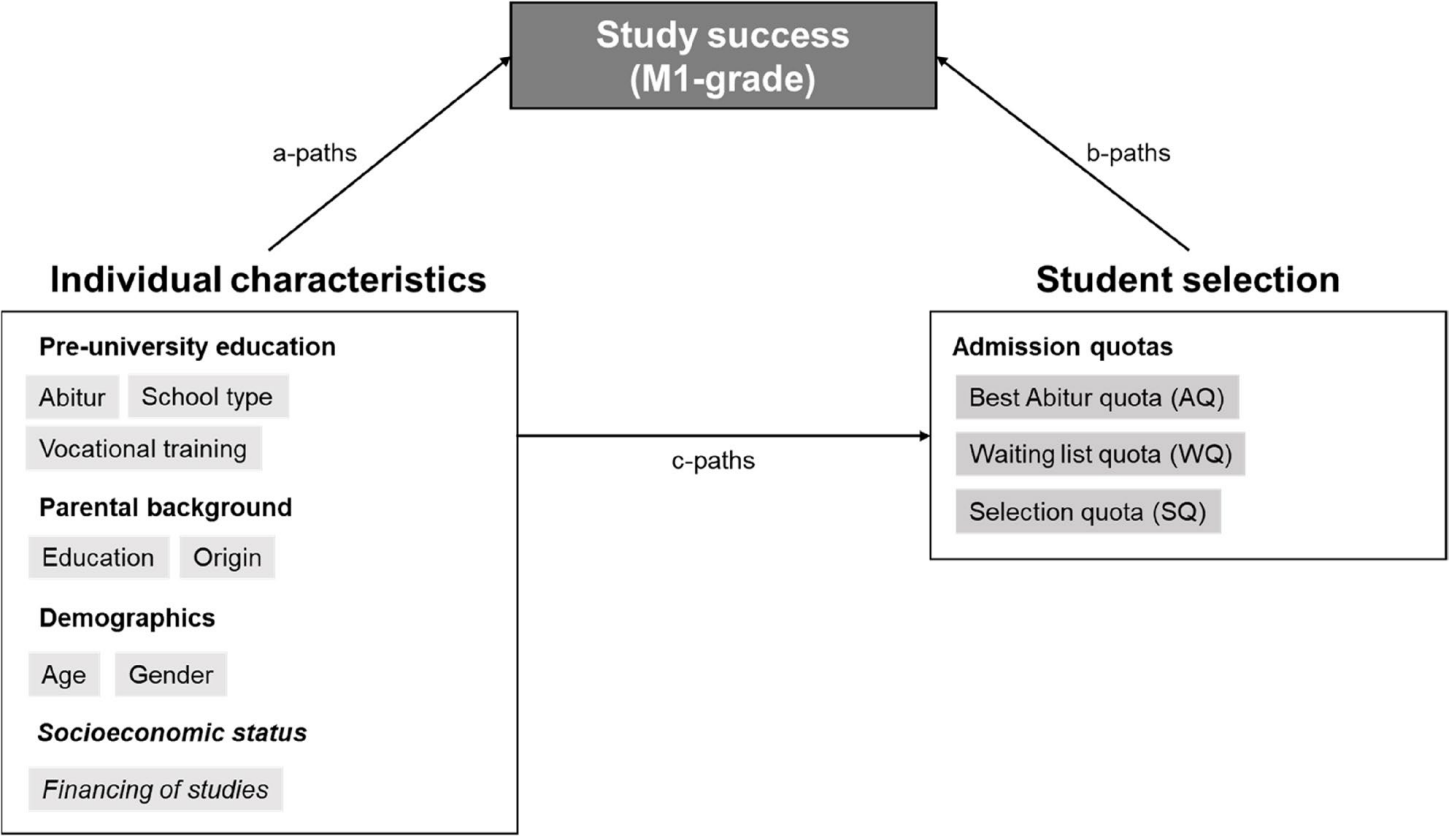
Conceptual path model of direct effects on study success and student selection. Notes: Schematic representation of the structural equation model (SEM) with observed variables. Individual characteristics directly influence study outcomes (M1, grade, a-paths) and admission routes (c-paths). The latter also directly affect study success (b-paths), and act as a mediator for the effect of individual characteristics on study success (c×b). In the SEM, individual characteristics are temporally ordered components of educational trajectories. This part of the SEM is sketched in Fig. 2. “Financing of studies” is in italics, because it is modeled as directly influencing study outcomes, but not admission routes

Our analytic strategy combines a structural equation model (SEM) of observed variables with a mediation decomposition of estimated effects. As illustrated in Fig. 1, we model academic success to be directly influenced by the set of observed individual characteristics (a-paths) as well as by the admission route through which students enter medical school (b-paths). In addition, individual characteristics are allowed to influence admission outcomes directly (c-paths), reflecting the fact that selection procedures draw on prior achievement and biographical information.

As indicated by the c-paths in Fig. 1, we model individual characteristics as predictors of admission outcomes. Admission routes, in turn, act as mediators of the effects of individual characteristics on academic success. For example, secondary school performance, a proxy for cognitive ability, affects M1 performance directly (a-path), but also indirectly through its influence on the probability of admission via specific selection routes (c × b).

A key feature of our analytical approach is that individual characteristics are not treated as independent predictors or mere control variables. Instead, they are conceptualized as temporally ordered and causally connected components of an educational trajectory. As a consequence, many individual characteristics function not only as predictors but also as (often multiple) mediators within the model.

**Fig. 2:**
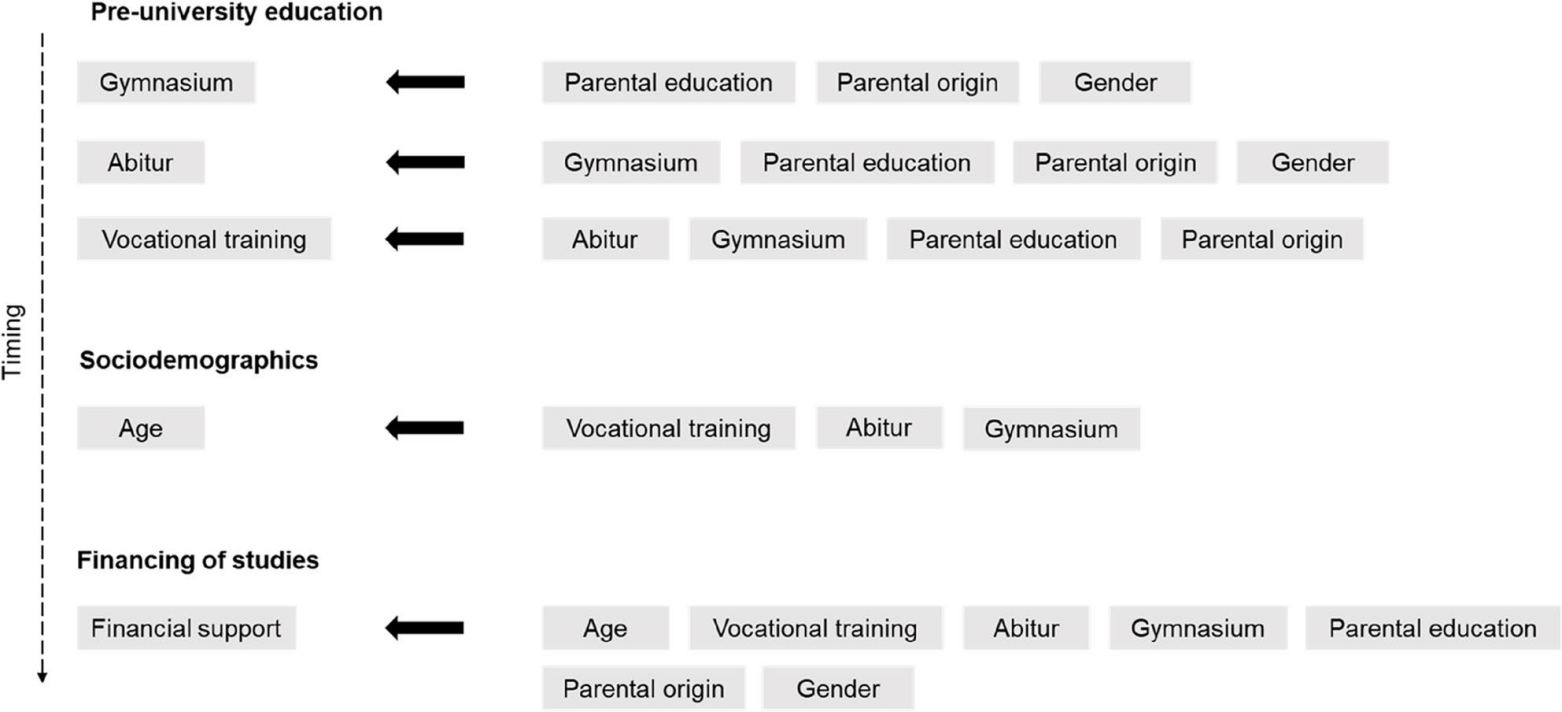
Conceptual path model for directed influences of individual characteristics. *Notes*: In the SEM, the left-hand factors are directly influenced by the right-hand variables. The SEM includes a temporal ordering of variables. Parental background and gender are not influenced by other characteristics. The SEM indicates a larger number of indirect effects, with individual characteristics functioning as (multiple) mediators themselves. E.g., gender affects the age at enrollment via school type and Abitur grade

The direct relationships among these characteristics are schematically depicted in Fig. 2. Parental background and gender are treated as exogenous variables, fixed at birth or at the outset of the educational pathway. They influence subsequent educational decisions and outcomes but are not themselves affected by later variables. The choice of secondary school precedes and shapes school achievement, which is central for direct access to medical school. When school performance is insufficient for immediate admission, applicants often pursue vocational training and employment during waiting periods, particularly as professional experience has become an explicit selection criterion. These pathways are associated with older age at enrolment and may also affect students’ financial dependence on their families once enrolled.

Beyond the direct paths shown in Fig. 2, the model implies numerous longer causal chains in which individual characteristics operate as mediators. For instance, we do not assume a direct causal effect of gender on vocational training choices. Instead, gender influences school performance, which in turn affects the likelihood of vocational training, admission route, age at enrolment, and ultimately academic success. Similarly, parental education affects school type and achievement, which shape admission probabilities, subsequent educational trajectories, and financial circumstances during study. For exogenous variables such as gender or parental background, but also for secondary education, indirect effects on academic success therefore operate through multiple sequential mediators.

As a result, the SEM generates a large number of indirect pathways linking individual characteristics to admission outcomes and academic performance. Rather than reporting each individual path, we present aggregated indirect effects in the results section, defined as the sum of all indirect pathways implied by the model.

### Statistical analysis

The structural equation model (SEM) outlined in Figs. 1 and 2 was specified exclusively with observed variables and estimated by maximum likelihood. All path coefficients from the SEM are reported in standardized form, allowing them to be interpreted as correlations and facilitating comparison across variables measured on different scales. This standardization serves descriptive and explanatory purposes within the SEM framework and reflects the relative strength of associations rather than marginal effects on an outcome scale. Admission routes are represented in the model by two indicator variables for WQ and SQ, while AQ serves as the reference category. This specification was chosen because the three routes are mutually exclusive and collectively exhaustive in our data. Interpreting effects relative to AQ is also substantively meaningful, as this route represents the academically most selective pathway based primarily on prior school achievement. Complete SEM results, including all standardized path coefficients, standard errors, and fit indices, are reported in Tables A1 and A2 in the Appendix.

While the SEM provides a coherent representation of the overall dependency structure, standardized path coefficients alone are not sufficient to quantify how much a given characteristic ultimately affects downstream outcomes such as admission probabilities or academic performance. We therefore complemented the SEM with a mediation analysis that decomposes effects into direct and indirect components. This decomposition aggregates all causal pathways implied by the SEM, including multi-step mediation chains involving both individual characteristics and admission routes.

In contrast to the standardized SEM coefficients, mediation effects are expressed on the natural metric of the outcome variable and can be interpreted as average changes in the outcome associated with a one-unit change in the predictor. For example, an effect on age at enrolment reflects the expected change in years associated with a one-point difference in the Abitur grade (e.g., from “A” to “B”). These effect estimates therefore capture substantively meaningful “level changes” rather than standardized associations. Effects of covariates on WQ and SQ should be interpreted relative to the AQ pathway. Likewise, effects of WQ and SQ on M1 grades represent differences in expected academic outcomes compared with students admitted through AQ, holding other characteristics constant. Standard errors for direct, indirect, and total effects (i.e., the sum) were derived using asymptotic approximations based on the delta method, allowing statistical inference for both individual pathways and aggregated effects.

To further substantiate the presence and relevance of mediation, we additionally report the proportion mediated (PM), defined as the ratio of the indirect effect to the total effect. This statistic serves two complementary purposes. First, it provides an interpretable measure of how much of an overall association operates through indirect pathways rather than directly. Second, it constitutes a formal statistical assessment of mediation, addressing whether indirect pathways account for a non-trivial and statistically distinguishable share of the total effect.

In addition to reporting SEM fit indices in the main text and extended supplementary materials, we conducted several sensitivity analyses to assess the robustness of our findings. Alternative outcome measures for academic performance included: (1) a binary indicator equal to one if a student’s M1 was equal to one (‘A’), and (2) time to M1 completion in years (with two years representing the standard curriculum duration). We further examined whether replacing the indicator for family financial support with a binary measure of student employment during medical school altered the results.

All statistical analyses were conducted using Stata 14 (StataCorp, College Station, TX). In the text, we refer to coefficients or group differences as statistically significant, when *p* < 0.05.

## Results

### Descriptive sample statistics

Table 1 presents descriptive statistics for all individual characteristics included in the analytical model (see Fig. 1), stratified by the admission routes and academic performance (average M1-grade).

**Table 1:** Descriptive sample statistics

		Admission quotas			Average M1-grade		
	Full sample (*n* = 644)	AQ (*n* = 144)	WQ (*n* = 84)	SQ (*n* = 416)	Yes	No	*P* =
Female	71.27% (459)	67.36% (97)	65.4% (55)	73.80% (307)	1.88 ± 0.62 (459)	1.80 ± 0.65 (185)	0.129
Gymnasium	83.70% (539)	88.89% (128)	82.14% (69)	82.21% (342)	1.81 ± 0.62 (539)	2.11 ± 0.63 (105)	*< 0.001*
Voc. Training	31.83% (205)	9.03% (13)	100.00% (84)	25.96% (108)	2.01 ± 0.65 (205)	1.78 ± 0.61 (439)	*< 0.001*
Mother born in Germany	87.58% (564)	86.11% (124)	88.10% (74)	87.98% (366)	1.83 ± 0.64 (564)	2.06 ± 0.56 (80)	*0.002*
Father born in Germany	86.49% (557)	84.03% (121)	83.33% (70)	87.98% (366)	1.84 ± 0.64 (557)	1.97 ± 0.56 (87)	0.067
Mother academic	46.58% (300)	53.47% (77)	32.14% (27)	47.12% (196)	1.81 ± 0.62 (300)	1.89 ± 0.64 (344)	0.107
Father academic	52.80% (340)	56.25% (81)	36.90% (31)	54.81% (228)	1.81 ± 0.63 (340)	1.91 ± 0.63 (304)	*0.038*
Financial support	64.13% (413)	75.69% (109)	20.24% (17)	68.99% (287)	1.81 ± 0.65 (413)	1.94 ± 0.59 (231)	*0.006*
Age (in years)	21.16 ± 3.20	19.61 ± 1.55	27.09 ± 2.68	20.50 ± 2.28			
Abitur grade	1.45 ± 0.47	1.05 ± 0.19	2.40 ± 0.47	1.41 ± 0.23			
M1-grade	1.86 ± 0.63	1.51 ± 0.57	2.17 ± 0.56	1.91 ± 0.62			

Summary statistics for all variables used in this study. Percentages and raw counts in parentheses for binary indicators, Mean ± SD for continuous and categorical variables. Calculations based on *N* = 644 survey respondents whose M1-grade (∊ [1,2,…,5]) was ≤ 3 and who were admitted via the selection quotas AQ (best school graduates), WQ (waiting list quota; since 2020: special aptitude quota for professionally experienced applicants), or SQ (MHH selection quota). The M1 failure grade 5 and the worst passing grade 4 are not included because of a very few cases. grade: 1.0 (best) to 4.0 (worst passing grade). The average M1-grade is stratified by the state of the binary indicators, thus 0/1. Statistical significance of differences between M1-grades is tested with two-sided Mann-Whitney *U*-tests. *P*-values < 0.05 are in italics

Clear differences in cohort composition emerge across selection quotas. Students admitted via the top school leavers quota (AQ) typically required near-perfect Abitur grades to enter medical school directly after secondary education. The average Abitur grade among students admitted through the university-specific selection quota (SQ) is lower, while students admitted via the waiting-time/special aptitude quota (WQ) show substantially weaker school performance on average. When direct entry into medical school is precluded by lower school grades, admission is commonly achieved through an alternative pathway involving waiting time and, in most cases, prior vocational training in a medical field. All WQ students in the sample followed such a pathway, and vocational training has also become an increasingly relevant selection criterion within SQ, where approximately one quarter of students report a completed medical vocational qualification.

These different educational trajectories are closely associated with age at enrolment. Students admitted via WQ are, on average, considerably older than those in AQ and SQ, reflecting periods of waiting time, training, and employment prior to admission. Differences in life-course trajectories are also mirrored in patterns of study financing. While around 64–76% of students in AQ and SQ report primary financial support from their parents, this proportion drops to approximately 20% among WQ, for whom employment, savings, or student loans are more common sources of funding. Admission routes further differ in terms of parental education, but not in terms of parental migration background: in AQ and SQ, more than half of students report that at least one parent holds a university degree, compared with roughly one third in WQ.

Differences between admission routes persist into academic performance. Students admitted via AQ achieve significantly better average M1 grades than those in SQ (*p* < 0.001, Mann-Whitney *U*-test), who in turn outperform students admitted via WQ (*p* = 0.001). Nevertheless, academic success is not confined to any single route: WQ students also achieve a solid “good” M1-grade on average.

Poorer M1-grades are moderately correlated with lower school performance (*r* = 0.29, *p* < 0.001) and higher age at enrolment (*r* = 0.26, *p* < 0.001). With respect to the binary individual characteristics in Table 1, students who attended a Gymnasium, whose mother was born in Germany, whose father holds a university degree, and who receive family financial support, have more academic success (Mann-Whitney *U*-test). Conversely, vocational training prior to enrolment is associated with a higher M1-grade.

Importantly, many of the observed associations reflect the underlying educational and life-course trajectories sketched in Figs. 1 and 2 rather than direct influences on academic performance. For example, the absence of parental financial support is not necessarily linked to poorer performance per se, but is closely tied to age at enrolment, prior employment, and family responsibilities; thus, factors that themselves emerge from earlier educational decisions and constraints.

The structural equation model and accompanying mediation analysis allow us to disentangle direct from indirect associations by explicitly modelling the temporal and causal ordering of individual characteristics, admission routes, and academic outcomes.

### Mediation analysis

#### Direct effects on study success and student selection

Table 2 presents the direct effects of individual characteristics on study success and admission routes (corresponding to the *a* and *c* paths in Fig. 1), as well as the direct, unmediated effects of the admission routes on academic performance (the *b* paths in Fig. 1), as specified in the SEM.

**Table 2:** Direct effects of individual characteristics andstudent selection on study success

	(1)	(2)	(3)
	M1-grade	WQ	SQ
Gymnasium	-0.241	0.086	-0.216
	(0.066, 0.000)	(0.022, 0.000)	(0.051, 0.000)
Abitur grade	0.224	0.350	0.184
	(0.093, 0.017)	(0.025, 0.000)	(0.056, 0.000)
Voc. Training	-0.132	0.001	0.047
	(0.074, 0.075)	(0.025, 0.954)	(0.057, 0.407)
Gender	0.080	-0.016	0.096
	(0.051, 0.113)	(0.017, 0.344)	(0.039, 0.014)
Age	0.032	0.041	-0.072
	(0.014, 0.021)	(0.004, 0.000)	(0.010, 0.000)
Mother German	-0.276	0.031	-0.049
	(0.089, 0.002)	(0.030, 0.308)	(0.069, 0.476)
Father German	0.027	-0.044	0.120
	(0.086, 0.751)	(0.029, 0.132)	(0.066, 0.069)
Mother academic	-0.031	0.018	-0.032
	(0.052, 0.543)	(0.018, 0.314)	(0.040, 0.430)
Father academic	-0.035	-0.001	0.027
	(0.051, 0.496)	(0.017, 0.954)	(0.040, 0.492)
Financial support	0.065		
	(0.055, 0.238)		
Selection quotas (ref.: AQ)			
WQ	0.247		
	(0.147, 0.093)		
SQ	0.298		
	(0.064, 0.000)		

Reported coefficients represent direct effects of individual characteristics on selection quotas and study outcomes (see Fig. 1), estimated within the SEM framework using maximum likelihood. Standard errors (SE), *p*-values are in parentheses. SE are based on the observed information matrix (OIM). Direct effects quantify associations not operating through other individual characteristics included as mediators. See Tables A1 & A2 in the Appendix for full SEM results with standardized coefficients. All coefficients represent linear effects in original units; for binary dependent variables, coefficients indicate changes in the probability of the outcome occurring, expressed in percentage points, per one-unit increase or status-change (yes/no) in the predictor. For WQ and SQ, effects are measured relative to the reference group AQ

As expected, secondary school performance in column (1) is a strong predictor of academic success in medical school. A one-point deterioration in the final school grade is associated with an increase of about 0.22 points in the M1 grade. Students who obtained their school-leaving certificate at a Gymnasium perform significantly better than those graduating from other school types.

Completion of vocational training prior to enrolment is, in comparison to Table 1, only weakly associated with better study outcomes once we account for less favourable school grades.

Parental background variables show little direct relevance for academic performance. Neither parental education nor financial support from the family is directly associated with study success. The only exception concerns maternal origin: having a mother of German origin is associated with moderately better performance, whereas no comparable effect is observed for paternal origin. We also do not find an effect of gender on academic performances at medical school.

Although students admitted through AQ and SQ appear broadly similar in descriptive terms (see Table 1), the results in column (1) of Table 2 further indicate that admission through SQ is associated with a distinct and statistically significant direct disadvantage in academic performance. Students admitted via SQ, where cognitive criteria receive less weight relative to factors such as prior vocational experience or documented social engagement, achieve M1 grades that are on average 0.3 points lower than those admitted through AQ, after accounting for all direct and indirect influences on academic success included in the SEM.

For WQ, we observe a coefficient of similar magnitude. However, the difference relative to AQ does not reach statistical significance (*p* = 0.09), which likely reflects the comparatively small number of students admitted through this pathway.

Columns (2) and (3) of Table 2 report the direct effects of individual characteristics on admission via WQ and SQ, relative to the reference group AQ. School grades play the expected role: poorer grades substantially reduce the likelihood of admission through the primary grade-based route (AQ) in favour of SQ and especially WQ. Holding a Gymnasium degree increases the probability of admission through AQ rather than SQ.

Age at enrolment and gender show very small but mostly statistically significant direct effects on admission probabilities. Parental origin and education show no systematic associations with admission routes.

#### Direct relationships among individual characteristics (educational pathways)

Table 3 reports the direct relationships among the individual characteristics included in the SEM, corresponding to the pathways specified in Fig. 2.

**Table 3:** Direct effects on individual characteristics

	(1)	(2)	(3)	(4)	(5)
	Gymnasium	Abitur	Vocational Training	Age	Financial support
Gymnasium		-0.016	-0.215	-1.350	0.010
		(0.050, 0.741)	(0.039, 0.000)	(0.196, 0.000)	(0.047, 0.824)
Abitur			0.584	3.030	0.015
			(0.031, 0.000)	(0.019, 0.000)	(0.052, 0.773)
Voc. Training				3.004	-0.154
				(0.195, 0.000)	(0.053, 0.003)
Gender	0.013	-0.061			-0.029
	(0.032, 0.682)	(0.041, 0.134)			(0.036, 0.421)
Age					-0.044
					(0.009, 0.000)
Mother German	-0.006	-0.023	-0.035		0.164
	(0.057, 0.918)	(0.071, 0.749)	(0.055, 0.525)		(0.064, 0.010)
Father German	0.058	-0.064	0.078		0.157
	(0.054, 0.281)	(0.069, 0.352)	(0.053, 0.143)		(0.061, 0.010)
Mother academic	0.021	-0.110	-0.046		0.069
	(0.033, 0.522)	(0.041, 0.008)	(0.032, 0.150)		(0.037, 0.061)
Father academic	0.032	-0.093	-0.004		0.120
	(0.032, 0.324)	(0.041, 0.023)	(0.032, 0.895)		(0.036, 0.001)

Coefficients represent direct effects of individual characteristics on other individual characteristics (see Fig. 2), estimated within the SEM framework using maximum likelihood. Standard errors (SE), *p*-values are in parentheses. SE are based on the observed information matrix (OIM). Direct effects quantify associations not operating through other individual characteristics included as mediators. See Tables A1 & A2 in the Appendix for full SEM results with standardized coefficients. All coefficients represent linear effects in original units; for binary dependent variables, coefficients indicate changes in the probability of the outcome occurring, expressed in percentage points, per one-unit increase or status-change (yes/no) in the predictor

While we do not find any influences on school choice, our results show that the educational level of both mother and father has a small but statistically significant association with better school performance. Column (3) focuses on vocational training prior to enrolment. A one-point deterioration in the Abitur grade significantly increases the probability of having completed vocational training by more than 50%. Graduates from Gymnasium are substantially less likely to report vocational training.

The educational pathway is also strongly associated with age at enrolment (column (4)): Gymnasium graduates are, on average, almost 1.5 years younger at the start of medical school than graduates from other secondary school types. Both poorer school performance and prior vocational training are also associated with a significantly higher age at enrolment. This pattern reflects the institutional design of the admission system, where weaker school grades often necessitate indirect educational pathways involving waiting periods and professional training.

Finally, column (5) of Table 3 reports direct determinants of financial support during medical school. Both parental origin and parental education are positively associated with the likelihood of receiving financial support from the family, although effect sizes are modest. In contrast, higher age at enrolment and prior vocational training are directly associated with a lower probability of family-based financial support.

#### Indirect effects

The direct effects reported above describe how individual characteristics, admission routes, and academic performance are linked conditional on the full model. However, the structure of the SEM explicitly allows these characteristics to operate as mediators themselves along temporally ordered educational trajectories. Table 4 therefore reports the aggregated indirect effects implied by Figs. 1 and 2, summarizing how individual characteristics influence outcomes through all admissible causal chains in the model.

**Table 4:** Indirect effects on study success, student selection, and individual characteristics

	(1)	(2)	(3)	(4)	(5)	(6)	(7)
	M1	WQ	SQ	Abitur	Vocational Training	Age	Financial Support
Gymnasium	-0.056	-0.091	0.137		-0.010	-0.725	0.126
	(0.034, 0.098)	(0.030, 0.002)	(0.024, 0.000)		(0.029, 0.741)	(0.268, 0.007)	(0.025, 0.000)
Abitur	0.015	0.197	-0.320			1.755	-0.300
	(0.081, 0.062)	(0.020, 0.000)	(0.043, 0.000)			(0.146, 0.000)	(0.040, 0.000)
Voc. Training	0.056	0.123	-0.219				-0.132
	(0.041, 0.731)	(0.015, 0.000)	(0.033, 0.000)				(0.029, 0.000)
Gender	-0.004	-0.033	0.007	0.000	-0.038	-0.318	0.019
	(0.021, 0.861)	(0.022, 0.134)	(0.007, 0.271)	(0.008, 0.797)	(0.025, 0.122)	(0.205, 0.122)	(0.013, 0.129)
Age	-0.014						
	(0.006, 0.013)						
Mother German	0.017	0.008	0.003	0.000	-0.015	0.016	0.003
	(0.038, 0.660)	(0.040, 0.837)	(0.014)	(0.001, 0.922)	(0.025, 0.737)	(0.398, 0.968)	(0.027, 0.919)
Father German	-0.012	-0.026	-0.009	-0.001	-0.050	-0.193	0.004
	(0.037, 0.750)	(0.038, 0.504)	(0.015, 0.532)	(0.003, 0.752)	(0.042, 0.230)	(0.383, 0.614)	(0.026, 0.884)
Mother academic	-0.044	-0.066	0.021	0.000	-0.069	-0.707	0.047
	(0.022, 0.047)	(0.023, 0.004)	(0.010, 0.028)	(0.001, 0.769)	(0.025, 0.007)	(0.230, 0.002)	(0.016, 0.003)
Father academic	-0.029	-0.050	0.009	0.001	-0.061	-0.498	0.030
	(0.022, 0.198)	(0.023, 0.026)	(0.009, 0.299)	(0.002, 0.754)	(0.025, 0.014)	(0.228, 0.029)	(0.016, 0.061)

Indirect effects quantify associations between variables that operate through one or more mediating pathways specified in the structural model (see Figs. 1 and 2). They are calculated as the product of the corresponding path coefficients along each mediated pathway and aggregated where multiple indirect paths exist in the SEM. See Tables A1 & A2 in the Appendix for full SEM results with standardized coefficients. All coefficients represent linear effects in original units; for binary dependent variables, coefficients indicate changes in the probability of the outcome occurring, expressed in percentage points, per one-unit increase or status-change (yes/no) in the predictor. Effects for WQ and SQ are measured relative to the reference group AQ. Standard errors (SE), *p*-values are in parentheses. SE are based on the observed information matrix (OIM)

While strong direct effects of pre-university education on study success were observed, column (1) of Table 4 shows that these characteristics do not exert additional indirect effects on academic performance via downstream variables in the educational pathway. This applies to school type, Abitur grade, and vocational training as well as to all other individual characteristics included in the SEM.

In contrast, indirect effects play a more prominent role in student selection (columns (2) and (3)). School type, school performance, and vocational training exert sizeable indirect influences on admission probabilities, thus, selection via WQ or SQ instead of AQ. A poorer Abitur grade affects admission mainly through the pathway of vocational training and delayed enrolment, increasing the probability of admission via the waiting-time quota (WQ). Vocational training itself increases chances of admission via WQ (relative to AQ) and decreases admission via SQ: since no vocational training is preceded by better Abitur grades, achieved at a Gymnasium, admission via AQ is more likely compared to quotas where professional experience is explicitly bonified.

We additionally find small but partially significant indirect influences of parental characteristics, which again work through the channels described in Figs. 1 and 2.

Columns (4)–(7) of Table 4 show that age at enrolment is particularly shaped by indirect pathways. A one-point deterioration in the Abitur grade increases the likelihood of vocational training and precludes direct access to medical school, resulting in an indirect increase in age at enrolment of approximately 1.5 years. Having graduated from a Gymnasium decreases age at enrolment by almost one year. Higher parental education, both maternal and paternal, is also associated with earlier enrolment through school choice, school performance, and the reduced need for vocational detours.

As shown previously, higher age at enrolment and vocational training are associated with a lower probability of receiving financial support from the family. This mechanism is reflected in column (7): attending a Gymnasium and achieving better school grades increase the likelihood of family-based financial support primarily through earlier enrolment and avoiding vocational training.

#### The proportion of mediated effects

While Table 4 shows absolute indirect effects, these estimates do not indicate how important mediation is relative to the total association between a predictor and an outcome. Table 5 therefore reports the proportion mediated (PM), defined as the ratio of the aggregated indirect effect to the total effect. PM serves two purposes: first, as a formal test of mediation, and second, as an interpretive aid that quantifies the relative contribution of indirect pathways.

**Table 5:** Proportion mediated of total effects: estimates and statistical inference

	(1)	(2)	(3)	(4)	(5)	(6)	(7)
	M1	WQ	SQ	Abitur grade	Vocational Training	Age	Financial support
Gymnasium	0.188	17.465	-1.742		0.043	0.350	0.923
	(0.111, 0.091)	(114.67, 0.879)	(1.229, 0.157)		(0.124, 0.730)	(0.091, 0.000)	(0.322, 0.004)
Abitur grade	0.402	0.360	2.350			0.367	1.052
	(0.220, 0.068)	(0.036, 0.000)	(0.729, 0.001)			(0.029, 0.000)	(0.186, 0.000)
Voc. training	-0.745	0.988	1.277				0.461
	(0.865, 0.389)	(0.199, 0.000)	(0.404, 0.002)				(0.121, 0.000)
Gender	-0.049	0.669	0.061	0.004	1	1	-1.773
	(0.277, 0.859)	(0.278, 0.016)	(0.076, 0.427)	(0.014, 0.800)			(6.847, 0.796)
Age	-0.837						
	(0.739, 0.257)						
Mother German	-0.064	0.209	-0.075	0.004	-0.715	1	0.017
	(0.148, 0.664)	(0.824, 0.799)	(0.361, 0.836)	(0.044, 0.925)	(4.123, 0.862)		(0.157, 0.911)
Father German	-0.763	0.367	-0.086	0.015	-1.835	1	0.014
	(5.129, 0.882)	(0.383, 0.338)	(0.154, 0.574)	(0.050, 0.764)	(5.594, 0.743)		(0.161, 0.932)
Mother academic	0.586	1.366	-2.031	0.003	0.598	1	0.403
	(0.518, 0.259)	(0.525, 0.009)	(8.130, 0.803)	(0.011, 0.770)	(0.190, 0.002)		(0.155, 0.009)
Father academic	0.453	0.981	0.255	0.006	1.073	1	0.192
	(0.524, 0.387)	(0.331, 0.003)	(0.340, 0.454)	(0.018, 0.756)	(0.597, 0.072)		(0.097, 0.048)

The proportion mediated (PM) expresses the relative contribution of indirect pathways to the total association between predictors and outcomes. PM is calculated as the ratio of the indirect effect to the total effect (direct + indirect effect). Standard errors (SE), *p*-values in parentheses. SE are derived using the delta method, allowing statistical inference under asymptotic normality. Where no direct relation is modelled in the SEM, the PM is by default = 1. Blank cells indicate the absence of an indirect effect. Variables where no indirect effects are modelled are omitted in this table

A negative PM indicates that direct and indirect effects operate in opposite directions. PM values greater than one imply that indirect effects exceed the total effect, which occurs when direct and indirect components partially offset each other. Blank cells indicate that no indirect effect is modelled, while PM equals one by construction if no direct effect is specified.

Consistent with the results above, mediation plays a very limited role for academic performance (column (1)), implying that effects operate directly through pre-university education.

For student selection (columns (2) and (3)), mediation plays a substantially larger role. Approximately 40% of the total effect of a higher Abitur grade on admission through WQ (relative to AQ) operates indirectly via vocational training and age at enrolment. Likewise, the effect of prior vocational training on admission through WQ is almost entirely mediated by age at enrolment.

Gender differences in admission through WQ are also largely indirect (PM ≈ 0.67), indicating that much of the observed association operates through gender differences in prior educational pathways and vocational training rather than through a direct effect of gender itself. For parental education, the proportion mediated is close to or slightly above one and statistically significant, suggesting that the association with WQ admission is almost entirely explained by indirect pathways through earlier educational achievements and career decisions.

For admission through SQ, mediation effects for pre-university education are even more pronounced. The proportion mediated exceeds one for both the Abitur grade (PM ≈ 2.4) and vocational training (PM ≈ 1.3), indicating that indirect pathways dominate the relationship and that the remaining direct effects point in the opposite direction.

Indirect pathways are also relevant for intermediate outcomes within the model. For vocational training, around 60% of the association with maternal academic background is mediated through preceding educational variables, whereas the corresponding estimate for paternal education slightly exceeds one but does not reach statistical significance.

Age at enrolment itself is partly shaped by earlier educational decisions: roughly one third of the total effects of secondary school type and Abitur grade on age operate indirectly through vocational training. Finally, financial dependence on the family is almost entirely mediated by earlier educational variables in the case of school type and Abitur grade, while vocational training accounts for roughly half of its total effect. For parental education, smaller but statistically significant mediated proportions (around 0.2–0.4) indicate that part of the socioeconomic gradient in study financing emerges through earlier educational pathways.

Taken together, the mediation analysis confirms that indirect pathways are central for understanding student selection and educational trajectories, but play a limited role for academic performance once students are enrolled. The SEM thus disentangles where background characteristics shape outcomes directly and where their influence is transmitted through structured educational pathways.

### Goodness-of-fit and robustness checks

Table 6 summarizes the goodness-of-fit statistics for the structural equation model described in Sect. Study design and conceptual model. The χ^2^-test comparing the model with the saturated model is statistically significant, formally suggesting a deviation from perfect fit. However, given the known sensitivity of the χ^2^-test to larger sample sizes, alternative fit indices provide a more informative assessment. The RMSEA = 0.04 and a *pclose* of 0.76 indicate a close model fit. The SRMR is exceptionally low at 0.01, further supporting a well-fitting model. Comparative fit indices confirm the adequacy of the model; both CFI and TLI exceed common thresholds for acceptable to excellent model fit.

**Table 6:** Goodness-of-Fit statistics (SEM)

Fit statistic	Description	Value
Likelihood ratio		
χ^2^ ms(9)	model vs. saturated	14.629
* p* > χ^2^		0.067
χ^2^ bs(81)	baseline vs. saturated	2510.114
* p* > χ^2^		0.000
Population error		
RMSEA	Root mean squared error of approximation	0.036
90% CI, lower bound		0.000
upper bound		0.065
pclose	Probability RMSEA ≤ 0.05	0.763
Information criteria		
AIC	Akaike’s information criterion	9707.584
BIC	Bayesian information criterion	10047.129
Baseline comparison		
CFI	Comparative fit index	0.997
TLI	Tucker-Lewis index	0.977
Size of residuals		
SRMR	Standardized root mean squared residual	0.011
CD	Coefficient of determination	0.165

Test statistics for the SEM formulated in Sect. Study design and conceptual model For the full SEM results see Table A1 in the Appendix

The coefficient of determination (CD) indicates that the model explains approximately 16.5% of the variance in the endogenous variables. Although modest in magnitude, this is consistent with expectations for models of academic success in highly selected student populations and with limited variance in key outcomes such as standardized exam scores [57, 58]. Taken together, the model demonstrates a theoretically coherent and statistically good fit to the data.

SEM specifications using alternative indicators of academic achievement, (i) a binary variable equal to 1 for an M1 score of 1 and (ii) time-to-M1 in years, yielded coefficients with magnitudes and statistical significance largely unchanged from the main model (see Tables S1 and S2 in the online supplement). Model fit indices also remained essentially stable (Table S3).

## Discussion

### Direct effects on study success and student selection

Our study confirms the well-documented finding that prior school performance is a strong and robust predictor of achievement in the early, preclinical phase of medical school. In line with this literature, we observe a statistically robust direct effect of the Abitur grade on M1 performance. In addition, the type of secondary school attended matters: attending a Gymnasium (the academically oriented secondary school track in Germany) had a meaningful direct effect on medical school performance. This finding is likely specific to the German educational system and may reflect that gymnasia provide more extensive preparation in science-related and other propaedeutic subjects than comprehensive or vocationally oriented schools [59, 60]. 

Pre-university educational characteristics also show clear and systematic direct effects on admission routes. These effects are strongest in quotas that rely heavily on cognitive criteria. Relative to the top school leavers quota (AQ), the selection quota (SQ) shows a negative direct association with attendance of a Gymnasium and with the Abitur grade. This pattern reflects the institutional logic of the admission system. Applicants with particularly strong school records are disproportionately admitted through AQ, which allocates places almost exclusively on the basis of prior academic achievement. Conditional on this channel, SQ therefore draws relatively more often from applicants whose profiles are strengthened by additional criteria such as vocational experience or documented social engagement. As a result, the student pool in SQ is more heterogeneous with respect to prior academic preparation.

Parental background and financial support – used here as proxies for migration background and socioeconomic status – do not exert direct effects on either admission routes or academic performance. With respect to selection, this aligns with institutional design: none of the admission quotas explicitly selects on these characteristics. For study success, however, we observe a notable exception. Maternal nativity shows a positive and statistically significant association with M1 performance, whereas paternal nativity does not. A plausible explanation may be the combined role of the mother in early child-rearing and in transmitting German as a first language at home [61, 62]. Paternal nativity, in contrast, is primarily linked to a slightly higher probability of parental financial support, suggesting a more indirect connection through socioeconomic resources rather than academic performance itself.

In bivariate comparisons, older age at enrolment is a consistent predictor of lower academic performance. In our modelling strategy, age is treated as an endogenous component of the educational trajectory rather than as an isolated control variable. Once earlier educational choices and achievements are accounted for, age has a negligible impact on M1 performance. Direct effects on admission routes are similarly small after conditioning on these factors, even though age differences are institutionally embedded in the design of the quotas. Overall, these results indicate that it is not age per se, but the educational pathway leading to later enrolment – most notably prior school performance – that drives observed differences.

### Independent effects of admission routes

At the institution studied, admission routes appear largely aligned with criteria that also exhibit predictive validity for academic success. This alignment can be regarded as a necessary condition for an effective and unbiased selection system. Given that German medical school admissions differ primarily in the weighting of sub-criteria rather than in their fundamental structure, this observation is likely to generalize beyond the immediate context.

Nevertheless, our results confirm that admission routes can exert independent effects on academic performance when they place greater emphasis on criteria with lower predictive validity for preclinical achievement. In the present study, these effects are moderate and more pronounced for SQ (compared to AQ) than for WQ. Both quotas still incorporate cognitive elements (such as aptitude tests and, to varying degrees, school grades) which likely attenuate adverse effects. Prior vocational training may plausibly convey clinically relevant experience and strong motivation for medicine; however, within a largely theory-based, examination-driven preclinical curriculum, these attributes are only weakly reflected in grades.

Criteria such as civic or voluntary engagement are more difficult to justify in terms of direct relevance for academic performance. While intended to signal social maturity or broader life experience, their connection to the competencies assessed in early medical training remains intangible. Importantly, such criteria still reward applicant choices and achievements. This distinguishes them from mechanisms that adjust selection outcomes based on characteristics such as gender, ethnicity, or socioeconomic background.

In our data, these characteristics show no direct association with academic success. We therefore interpret our findings as a cautionary signal against overemphasizing selection criteria that are weakly linked to academic outcomes, and as further confirmation that cognitive criteria and domain-specific tests remain central predictors of medical school performance [7], which itself is associated with later clinical competence [63–65]. 

At the same time, there is growing evidence that structured instruments such as Multiple Mini-Interviews (MMIs) and Situational Judgment Tests (SJTs) can complement cognitive measures and outperform unstructured interviews or motivational statements in predictive validity [51, 55, 56, 66, 67]. Selection tools that broaden assessment while maintaining a clear link to relevant competencies therefore appear promising. By contrast, selection criteria that substantially depart from academic merit, such as quotas based on gender or ethnicity, remain difficult to justify from a performance-oriented perspective.

### Indirect effects on study success and student selection

The absence of strong direct effects of background characteristics does not imply that these factors are irrelevant. As hypothesized, their influence operates primarily through indirect pathways shaping students’ educational trajectories. With respect to academic performance, we find no meaningful indirect relations in our data. Because girls outperform boys at school, they can embark on more favourable educational pathways [34, 68, 69], including earlier enrolment and a higher likelihood of family support during studies. These mediated pathways account for a substantial share of the total gender effect of (not) being admitted through the unfavourable WQ pathway, but do not translate to performance differences in medical school. Intergenerational characteristics such as parental education and nativity do not exert indirect effects on study success itself, but they play a role in shaping admission routes. A mother’s academic degree increases the probability of a pathway leading to direct admission via quota relying on cognitive criteria. Higher parental education in general reduces the likelihood that access to medical school occurs only after vocational training and waiting time. This is consistent with research showing that parental engagement in education is crucial for children’s cognitive development and is more likely and more effective in highly educated families [70–75]. The slightly stronger maternal than paternal effect may reflect the persistent reality that mothers remain more deeply involved in child-rearing and schooling than fathers, regardless of their own occupational status [73, 76–78]. 

Parental background also increases the probability of financial support during studies, although, in line with prior mixed evidence [79–84], study financing itself shows no association with academic performance.

Taken together, these findings underscore that background characteristics matter primarily by structuring access to advantageous educational pathways rather than by affecting academic success directly.

From a broader perspective, this raises a central normative question: should characteristics such as socioeconomic status, gender, or migration background be incorporated into admission decisions, despite their lack of direct association with academic performance? A body of literature argues that persistent underrepresentation of certain social groups in medical education constitutes an equity problem that may warrant corrective action at the admission stage (e.g. [85–87]). From this perspective, adjusting selection outcomes is seen as a means of compensating for unequal opportunities accumulated earlier in the educational trajectory.

An alternative view, supported by our findings, is that selection into a demanding and responsible profession should primarily prioritize demonstrated academic readiness, while inequalities arising earlier in the educational pathway should be addressed upstream. Our results suggest that disparities in admission and academic success are largely mediated through prior school performance and educational trajectories, rather than through direct effects of socioeconomic or demographic background.

Many sources of inequality observed at the university level thus appear to originate before university entry, a point that lies beyond the causal scope of the present study. Interventions aimed at reducing disparities in school performance – such as targeted academic support for students from disadvantaged backgrounds or for boys, who lag behind girls in average school achievement – may therefore represent a more effective and equitable strategy than adjusting admission criteria at the point of university entry.

### Limitations

This study was conducted at a single medical school, limiting external validity. A multi-centre design across different institutions and disciplines would be highly desirable. Our results may serve as a stimulus for such future work.

One strength of our study is the linkage of administrative data with extended survey information. The trade-off is low participation in online surveys, reducing the effective sample size. Since 2021, survey responses can be linked to exam outcomes, but many students surveyed in 2023 or 2024 had not yet completed the first state exam (M1), thereby further reducing the sample size. In addition, medical students constitute a comparatively homogeneous and cognitively high-performing group. This limited variability affects several predictors and, in particular, the outcome measure of academic success, where most students achieve good or very good results. As a consequence, estimated effects should be interpreted in light of the restricted variance.

A natural extension would be to estimate the SEM up to the second state exam (M2, five years into the program). However, data availability is limited, and the long time lag between school, application, and M2 complicates causal inference, as many intervening experiences accumulate.

To maintain clarity and interpretability, our SEM focused on key demographic and socioeconomic indicators. Data availability constrained the operationalization of socioeconomic status: detailed information on parental occupation, income, or wealth, as well as students’ own financial resources (e.g., earnings or assets), was not available in the survey. SES is therefore captured through a limited set of proxies, such as parental education and financial support during studies. Of course, many other factors may influence academic success. For example, while we analysed the motivation module of the HSM questionnaire separately, we found little association with individual characteristics or exam performance (not reported in this article). In medical education, this is unsurprising: due to both self-selection and the rigors of admission, students typically report consistently high levels of motivation throughout their studies (Tsikas and Fischer: Dimensions of medical students’ motivation and their stability over time: longitudinal evidence from a German panel data survey, submitted) [88, 89]. 

Although the SEM is specified using directed paths that reflect plausible temporal ordering and theoretically motivated causal assumptions, the analysis is based on observational data. The reported effects should therefore be interpreted as conditional associations consistent with the specified model rather than as definitive causal estimates.

For reasons of model complexity and interpretability, we did not estimate interaction effects (e.g., between gender and parental background or admission routes). While such interactions may be substantively relevant, incorporating them would substantially increase the number of paths and complicate the mediation structure, and thus falls beyond the scope of the present analysis.

We focused on academic performance because it is a well-established and operationalizable outcome that allows the effects of admission pathways, SES, and cognitive ability to be quantified with precision. As more comprehensive data on professional and non-academic competencies become available, future research could extend this framework to examine how these broader outcomes are shaped by selection processes and student background.

## Conclusion

This study examined how prior educational achievement, family background, and admission pathways jointly shape access to medical school and early academic performance. Using a structural equation model that integrates both direct and indirect relationships, we show that prior school performance remains the most consistent and robust predictor of success in the preclinical phase of medical training.

Admission pathways largely align with this pattern. Quotas that rely strongly on cognitive criteria select students who also perform well academically. At the same time, admission routes that place greater emphasis on additional criteria such as vocational experience or non-academic achievements generate a more heterogeneous student pool with respect to prior academic preparation. In our data, this is reflected in a moderate but statistically detectable difference in early examination performance between admission routes.

Importantly, demographic and socioeconomic background characteristics do not exert direct effects on academic success in our model. Their influence operates primarily through earlier educational pathways, particularly through school performance and the timing of university entry. This suggests that inequalities observed at the level of university admissions are largely rooted in processes occurring earlier in the educational trajectory.

From a policy perspective, these findings highlight a central trade-off in medical school admissions. Selection systems that prioritize academic readiness appear well aligned with the demands of the early curriculum. At the same time, concerns about representation and fairness may call for broader evaluation criteria. Our results suggest that policies aiming to reduce social disparities in access to medical education may be more effective if they address unequal educational opportunities before university entry rather than primarily modifying admission criteria at the point of selection.

## Supplementary Information

Supplementary Material 1: Appendix.

Supplementary Material 2.

## Data Availability

The data analysed during this study is not publicly available due to its proprietary nature and privacy concerning administrative data used for this article. Data are available from the corresponding author upon reasonable request.

## Abbreviations

AQ: Best abitur quota
CD: Coefficient of determination
CFI: Comparative fit index
GPA: Grade point average
HSM: Hannover Screening of Study Motivation
M1, M2: First & second part of the German national licensing exam
MHH: Hannover Medical School
MMI: Multiple Mini Interview
OSCE: Objective Structured Clinical Examination
PM: Proportion mediated
RMSEA: Root mean squared error of approximation
SEM: Structural equation model
SES: Socioeconomic status
SJT: Situational Judgment Test
SQ: Selection quota
SRMR: Standardized root mean squared residual
TLI: Tucker-Lewis index
WQ: Waiting list quota
