# Supplementary material for: Student background, admission routes, and academic success: a structural mediation analysis
Source: BMC Med Educ. 2026 Mar 24;26:578. doi: 10.1186/s12909-026-09068-z (PMC13064409; doi:10.1186/s12909-026-09068-z)
Supplement: Supplementary file 1 — Supplementary Material 1: Appendix. [file 12909_2026_9068_MOESM1_ESM.docx]

# **Appendix**

| **Table A1.** SEM output for study success and admission quotas | | | |
| --- | --- | --- | --- |
|  | (1) | (2) | (3) |
|  | **M1** | **WQ** | **SQ** |
| **Admission quotas** |  |  |  |
| WQ | 0.132 |  |  |
|  | (0.078, 0.092) |  |  |
| SQ | 0.226 |  |  |
|  | (0.048, 0.000) |  |  |
|  |  |  |  |
| **Pre-university education** |  |  |  |
| Gymnasium | -0.141 | 0.094 | -0.167 |
|  | (0.039, 0.000) | (0.024, 0.000) | (0.039, 0.000) |
| Abitur grade | 0.168 | 0.493 | 0.182 |
|  | (0.070, 0.017) | (0.034, 0.000) | (0.056, 0.001) |
| Vocational training | -0.097 | 0.002 | 0.046 |
|  | (0.054, 0.075) | (0.035, 0.954) | (0.056, 0.412) |
|  |  |  |  |
| **Sociodemographics** |  |  |  |
| Gender | 0.057 | -0.022 | 0.091 |
|  | (0.036, 0.112) | (0.023, 0.343) | (0.037, 0.013) |
| Age | 0.160 | 0.390 | -0.485 |
|  | (0.069, 0.021) | (0.041, 0.000) | (0.063, 0.000) |
|  |  |  |  |
| **Parental background** |  |  |  |
| Mother German | -0.144 | 0.030 | -0.034 |
|  | (0.046, 0.002) | (0.030, 0.308) | (0.047, 0.476) |
| Father German | 0.015 | -0.044 | 0.086 |
|  | (0.046, 0.751) | (0.029, 0.132) | (0.047, 0.068) |
| Mother academic | -0.025 | 0.026 | -0.033 |
|  | (0.041, 0.543) | (0.026, 0.313) | (0.041, 0.492) |
| Father academic | -0.028 | -0.001 | 0.028 |
|  | (0.041, 0.496) | (0.026, 0.954) | (0.041, 0.492) |
|  |  |  |  |
| **Financing of studies** |  |  |  |
| Family financial support | 0.049 |  |  |
|  | (0.042, 0.237) |  |  |
|  |  |  |  |
| **Var (ε)** |  |  |  |
| M1 | 0.831 (0.027) | | |
| WQ | 0.340 (0.022) | | |
| SQ | 0.873 (0.025) | | |
| Gymnasium | 0.994 (0.006) | | |
| Abitur grade | 0.963 (0.015) | | |
| Vocational training | 0.600 (0.030) | | |
| Age | 0.316 (0.021) | | |
| Family financial support | 0.747 (0.029) | | |
|  |  |  |  |
| **Cov. (ε.WQ*ε.SQ)** | -0.615 | | |
|  | (0.025, 0.000) | | |
| *Notes:* SEM results for study success (M1-grade) and admission quotas, see Fig. 1. See Table A2 for results on individual characteristics. Maximum Likelihood was used to estimate the model. Log likelihood = -4,777.792. N = 644. LR test (model vs. saturated): χ^2^ (8) = 14.63, *p* = 0.067. All coefficients are reported in standard deviations, alongside OIM standard errors and *p*-values in parentheses. See Tables 2-5 for a mediation analysis and Table 6 for goodness-of-fit statistics. | | | |

| **Table A2:** SEM-Output (cont’d) for individual characteristics | | | | | |
| --- | --- | --- | --- | --- | --- |
|  | (1) | (2) | (3) | (4) | (5) |
|  | **Gymnasium** | **Abitur grade** | **Voc. training** | **Age** | **Fin. support** |
| **Pre-university education** |  |  |  |  |  |
| Gymnasium |  | -0.013 | -0.171 | -0.156 | 0.008 |
|  |  | (0.039, 0.741) | (0.030, 0.000) | (0.024, 0.000) | (0.036, 0.825) |
| Abitur grade |  |  | 0.595 | 0.449 | 0.015 |
|  |  |  | (0.026, 0.000) | (0.029, 0.000) | (0.052, 0.778) |
| Voc. Training |  |  |  | 0.438 | -0.150 |
|  |  |  |  | (0.028, 0.000) | (0.051, 0.003) |
|  |  |  |  |  |  |
| **Sociodemographics** |  |  |  |  |  |
| Gender | 0.016 | -0.058 |  |  | -0.028 |
|  | (0.039, 0.682) | (0.039, 0.134) |  |  | (0.034, 0.421) |
| Age |  |  |  |  | -0.293 |
|  |  |  |  |  | (0.060, 0.000) |
|  |  |  |  |  |  |
| **Parental background** |  |  |  |  |  |
| Mother German | -0.005 | 0.016 | -0.025 |  | 0.113 |
|  | (0.051, 0.918) | (0.050, 0.352) | (0.039, 0.525) |  | (0.044, 0.009) |
| Father German | 0.054 | -0.046 | 0.057 |  | 0.112 |
|  | (0.050, 0.280) | (0.049, 0.352) | (0.039, 0.143) |  | (0.043, 0.010) |
| Mother academic | 0.028 | -0.115 | -0.050 |  | 0.072 |
|  | (0.044, 0.323) | (0.043, 0.007) | (0.034, 0.149) |  | (0.038, 0.060) |
| Father academic | 0.043 | -0.098 | 0.004 |  | 0.126 |
|  | (0.044, 0.323) | (0.043, 0.022) | (0.034, 0.895) |  | (0.038, 0.001) |
| *Notes:* SEM results (continued, see Table A1) for individual characteristics, see also Fig. 2. All coefficients are reported in standard deviations, alongside OIM standard errors and *p*-values in parentheses. See Tables 2-5 for a mediation analysis and Table 6 for goodness-of-fit statistics. | | | | | |
