# Supplementary material for: Student background, admission routes, and academic success: a structural mediation analysis
Source: BMC Med Educ. 2026 Mar 24;26:578. doi: 10.1186/s12909-026-09068-z (PMC13064409; doi:10.1186/s12909-026-09068-z)
Supplement: Supplementary file 2 — Supplementary Material 2. [file 12909_2026_9068_MOESM2_ESM.pdf]

Supplementary Materials

Table S1: SEM output for alternative study success measures

	M1-grade	OIM S.E.	*P=*	M1 = 1	OIM S.E.	*P=*	Study time	OIM S.E.	*P=*
WQ	0.132	0.078	0.092	-0.186	0.079	0.019	-0.082	0.081	0.311
SQ	0.226	0.048	0.000	-0.246	0.049	0.000	-0.048	0.051	0.349
Age	0.160	0.069	0.021	-0.075	0.071	0.288	0.308	0.071	0.000
Gymnasium	-0.141	0.039	0.000	0.111	0.039	0.005	-0.081	0.041	0.046
Abitur grade	0.168	0.070	0.017	-0.149	0.071	0.037	0.105	0.073	0.151
Voc. Training	-0.097	0.054	0.075	0.113	0.055	0.041	-0.056	0.057	0.329
Female	0.057	0.036	0.112	-0.067	0.037	0.069	0.058	0.038	0.125
Mother born in Germany	-0.144	0.046	0.002	0.120	0.047	0.010	-0.045	0.049	0.352
Father born in Germany	0.015	0.046	0.751	0.016	0.047	0.729	0.001	0.049	0.977
Mother academic	-0.025	0.041	0.543	0.008	0.041	0.389	0.052	0.043	0.220
Father academic	-0.028	0.041	0.496	0.035	0.041	0.389	0.030	0.043	0.475
Fin. Support: family	0.049	0.042	0.237	0.012	0.042	0.771	0.099	0.044	0.023
var(ε.M1)	0.831	0.027					0.905	0.022	
var(ε.WQ)	0.340	0.022					0.347	0.022	
var(ε.SQ)	0.873	0.025					0.876	0.025	
var(ε.Age)	0.316	0.021		0.316	0.021		0.322	0.021	
var(ε.Gymnasium)	0.994	0.006		0.994	0.006		0.994	0.006	
var(ε.Abitur grade)	0.963	0.015		0.963	0.015		0.963	0.015	
var(ε.Voc. Training)	0.600	0.030		0.600	0.030		0.605	0.300	
var(ε.Fin. Support: family)	0.747	0.029		0.747	0.029		0.746	0.029	
cov(ε.WQ*ε.SQ)	-0.615	0.025	0.000	-0.615	0.025	0.000	-0.613	0.025	0.000
N		644			644			638	

Notes: SEM results for the alternative study success measures "M1-grade = 1" (a binary variable that takes the value 1 if the M1 grade is = 1 (i.e. 'A')) and "Study time" until M1 was passed. The main results for the M1-grade indicator (see Table A2) are included for comparison. Maximum Likelihood was used to estimate the model. All coefficients are reported in standard deviations, alongside OIM standard errors and *p*-values. See Table S2 for the mediation analysis results and Table S3 for goodness-of-fit statistics.

Table S2: Mediation analysis for study success (alternative outcome measures)

	**M1 grade = 1**			**Study time M1**		
	Direct	Indirect	Total	Direct	Indirect	Total
WQ	-0.248		-0.248	-0.104		-0.104
	(0.106, 0.019)		(0.106, 0.019)	(0.103, 0.311)		(0.103, 0.311)
SQ	-0.230		-0.230	-0.042		-0.042
	(0.046, 0.000)		(0.046, 0.000)	(0.045, 0.349)		(0.045, 0.349)
Age	-0.011	0.006	-0.004	0.041	-0.005	0.036
	(0.010, 0.288)	(0.004, 0.150)	(0.009, 0.631)	(0.010, 0.000)	(0.004, 0.169)	(0.009, 0.000)
Gymnasium	0.136	0.021	0.156	-0.094	-0.058	-0.152
	(0.048, 0.005)	(0.023, 0.375)	(0.047, 0.001)	(0.047, 0.046)	(0.021, 0.006)	(0.045, 0.001)
Abitur grade	-0.141	-0.095	-0.236	0.094	0.089	0.182
	(0.053, 0.037)	(0.058, 0.104)	(0.036, 0.000)	(0.065, 0.150)	(0.056, 0.113)	(0.035, 0.000)
Vocational training	0.109	-0.027	0.082	-0.050	0.092	0.042
	(0.053, 0.042)	(0.030, 0.372)	(0.046, 0.076)	(0.052, 0.328)	(0.028, 0.001)	(0.045, 0.352)
Female	-0.066	-0.002	-0.068	0.055	-0.018	0.036
	(0.037, 0.070)	(0.014, 0.875)	(0.039, 0.076)	(0.035, 0.126)	(0.010, 0.087)	(0.036, 0.318)
Mother born in Germany	0.164	-0.004	0.160	-0.058	0.020	-0.039
	(0.064, 0.011)	(0.025, 0.882)	(0.068, 0.019)	(0.063, 0.352)	(0.018, 0.286)	(0.064, 0.550)
Father born in Germany	0.021	0.016	0.037	0.002	-0.004	-0.002
	(0.062, 0.729)	(0.024, 0.520)	(0.065, 0.571)	(0.060, 0.977)	(0.019, 0.844)	(0.062, 0.975)
Mother academic	0.007	0.029	0.036	0.044	-0.018	0.026
	(0.037, 0.846)	(0.015, 0.046)	(0.039, 0.356)	(0.036, 0.221)	(0.011, 0.111)	(0.037, 0.474)
Father academic	0.032	0.023	0.054	0.026	-0.013	0.012
	(0.037, 0.390)	(0.015, 0.046)	(0.039, 0.161)	(0.036, 0.036)	(0.011, 0.240)	(0.037, 0.740)
Fin. Support: family	0.012		0.012	0.088		0.088
	(0.040, 0.771)		(0.040, 0.771)	(0.039, 0.023)		(0.039, 0.023)

Notes: Direct, indirect and total effects for study success after SEM (Table S1). Table S2 presents the mediation analysis results for the alternative study success measures described in Table S1. OIM standard errors and p-values are in parentheses. All coefficients represent linear effects in original units; for binary dependent variables, coefficients indicate changes in the probability of the outcome occurring, expressed in percentage points, per one-unit increase or status-change (yes/no) in the predictor. Minor differences between direct + indirect and total effects may occur due to rounding errors. Blank cells indicate no paths between indicators. WQ: waiting list quota / special aptitude quota; SQ: MHH selection process.

Table S3: Goodness-of-Fit statistics (SEM) for alternative study success outcomes

Fit statistic	Description	M1 grade	M1 grade = 1	Study time M1
**Likelihood ratio**				
χ^2 ms(9)	model vs. saturated	14.629	14.629	13.554
$p > \chi^2$		0.067	0.067	0.094
χ^2 bs(81)	baseline vs. saturated	2,510.114	2,490.478	2,395.237
$p > \chi^2$		0.000	0.000	0.000
**Population error**				
RMSEA	Root mean squared error of approximation	0.036	0.036	0.033
90% CI, lower bound		0.000	0.000	0.000
upper bound		0.065	0.065	0.062
pclose	Probability RMSEA ≤ 0.05	0.763	0.763	0.806
**Information criteria**				
AIC	Akaike's information criterion	9,707.584	9,289.304	9,116.428
BIC	Bayesian information criterion	10,047.129	9,628.849	9,455.262
**Baseline comparison**				
CFI	Comparative fit index	0.997	0.997	0.998
TLI	Tucker-Lewis index	0.977	0.977	0.980
**Size of residuals**				
SRMR	Standardized root mean squared residual	0.011	0.011	0.011
CD	Coefficient of determination	0.165	0.164	0.153

Notes: Tests statistics for the SEM including the alternative measures for study success (see Table S1). The fit statistics of the main model (Table A1 and A2 in the Appendix, Table 6 in the main text) are included for comparison.
